# HMG Proteins from Molecules to Disease

**DOI:** 10.3390/biom12020319

**Published:** 2022-02-17

**Authors:** Aída Barreiro-Alonso, Ángel Vizoso-Vázquez, Mónica Lamas-Maceiras, María-Esperanza Cerdán

**Affiliations:** 1 EXPRELA Group, Centro de Investigaciones Científicas Avanzadas (CICA), Universidade da Coruña (UDC), 15008 A Coruña, Spain; aida.barreiro@udc.es (A.B.-A.); a.vizoso@udc.es (Á.V.-V.); monica.lamas@udc.es (M.L.-M.); 2 Instituto de Investigación Biomédica de A Coruña (INIBIC), 15006 A Coruña, Spain; 3 Department of Biology, Faculty of Sciences, Campus de A Zapateira, Universidade da Coruña (UDC), 15008 A Coruña, Spain

High Mobility Group (HMG) proteins are today the focus of interest due to their participation in human degenerative diseases and inflammatory responses. They participate in DNA repair, transcriptional regulation, and the epigenetic control of gene expression, but also in extranuclear and extracellular functions. They are highly conserved from yeasts to humans and they are classified into three families, HMGA, HMGB, and HMGN. Each one is recognized by a specific functional domain: the “AT hook” in HMGA, the “HMG-box” in HMGB, and the “nucleosomal binding domain” in HMGN proteins [1]. In this Special Issue “HMG Proteins from Molecules to Disease”, focused reviews and also original research papers are presented, gathering and relating recent advances in this field.

Starting with yeasts, Ángel Vizoso-Vázquez and coworkers [2] characterize a previously orphan protein from the non-conventional yeast *Kluyveromyces lactis*, which showed low sequence similarity to the HMGB protein Ixr1 from *Saccharomyces cerevisiae*, but that shares common functions, including its involvement in the response to hypoxia or oxidative stress induced by metals or hydrogen peroxide, as well as in the control of key regulators for maintenance of the dNTP pool and ribosome synthesis. However, *Kl*Ixr1’s and *Sc*Ixr1’s functions differ in cellular responses to cisplatin or cycloheximide in these yeasts.

In a feature paper, Helena Erlandsson Harris and collaborators [3] analyzed the role of different HMGB1 redox isoforms on the phenotypes of murine bone-marrow-derived macrophages (BMDMs). It is known that macrophages can be induced by HMGB1, and the authors demonstrate that stimulation with disulfide HMGB1 (dsHMGB1) or fully reduced HMGB1 (frHMGB1) induces cell migration. However, only dsHMGB1 induces a unique macrophage phenotype that secretes pro-inflammatory cytokines, and that differs from the classical proinflammatory macrophage phenotype, while frHMGB1 did not induce macrophage polarization. Michal Štros and coworkers [4] show experimental evidence of the participation of human HMGB1 and HMGB2 proteins in the pluripotency of human embryonic stem cells (hESCs) and their role in neuroectodermal cells, (hNECs). A thorough review presented by Jennifer F. Kugel and collaborators [5] summarizes recent advances in our understanding of the interactions of HMGB proteins with the genome and the impact on disease. Finally, the paper by Yansong Li and collaborators shows the correlation between blast injury and an early increase in HMGB1 plasma levels, along with severe tissue damage and high mortality; and the role of 2-O, 3-O desulfated heparin to inhibit local and systemic inflammatory responses [6].

Two reviews are focused on HMGA proteins. Josep Villanueva and collabora tors [7] discuss recent advances in the studies of high mobility group A1 (HMGA1) chromatin remodeling protein, as a chromatin regulator in cancer and stem cells; but also as a “moonlighting protein” with different functions depending upon cellular location. In particular, its functions in invasive triple-negative breast cancer (TNBC) cell lines when it is secreted and signals, through the receptor for advanced glycation end products (RAGE), to produce phenotypes involved in tumor invasion and metastasis. In a different stage, HMGA plays a pivotal role in successful pregnancy, and its dysfunction may be related to the pathogenesis of preeclampsia, a disease that can be fatal for the mother and fetus. Takashi Sugiyama and collaborators [8] review the role of HMGA protein in the pathogenesis of preeclampsia and its promising use as a reliable biomarker in this disease. They conclude that the molecular mechanism involved in this pathogenesis might be related to interference with extravillous trophoblasts invasion.

Finally, an extensive review on the role of mitochondrial HMG-box containing proteins in human diseases, selected as the editor’s choice, is presented by Ľubomir Tomáška and coworkers [9]. Mitochondrial DNA is compacted into nucleo-protein structures, called mitochondrial nucleoids, with the involvement of high-mobility group (HMG)-box containing proteins. Mitochondrial HMG-box proteins also protect mitochondrial DNA against damage, regulate gene expression and direct the segregation of DNA into daughter organelles. This paper overviews the structural basis of interaction with DNA, their functions, and how their defects lead to different pathologies.

In summary, this Special Issue highlights the role of HMG proteins in human diseases and anticipates their future importance in diagnosis or regenerative cellular therapies.
